# Descriptive Histology and Anatomy of the Nasal Cavity and Its Associated Sensory Organs in the European Hedgehog ( *Erinaceus europaeus* ) Based on Four Standardised Transverse Sections

**DOI:** 10.1111/ahe.70067

**Published:** 2025-10-19

**Authors:** Yannick Van de Weyer, Pietro Asproni, Steve Bexton, Valerie Tilston, Gail Leeming, Guido Rocchigiani

**Affiliations:** ^1^ Veterinary Anatomy, Physiology and Pathology Department Leahurst Campus, University of Liverpool Neston UK; ^2^ Tissue Biology and Chemical Communication Department IRSEA, Institute of Research in Semiochemistry and Applied Ethology Quartier Salignan France; ^3^ RSPCA East Winch Wildlife Centre East Winch UK

**Keywords:** conchae, olfactory, respiratory, steno's gland, vibrissae, vomeronasal organ

## Abstract

The nasal cavity of European hedgehogs ( *Erinaceus europaeus* ) harbours a well‐developed olfactory system, essential for food provision and communication. Additionally, it acts as a first line of defence by preventing pathogens and irritants from reaching the lungs, thereby playing an important physiological role. This study describes the histo‐anatomical features of the nasal cavity in the species. Hedgehogs have simple nasoturbinates and complex branching maxilloturbinates lined by respiratory epithelium. The caudal nasal cavity contains large lateral nasal glands and abundant olfactory epithelium, especially dorsally, lining most of the septum and ethmoid sinuses. The left and right nasal cavities merge at the nasopharynx.

## 1 Introduction

The European hedgehog ( *Erinaceus europaeus* ) is a nocturnal, insectivorous mammal that relies on its sense of smell for food provision, intraspecific communication and predator evasion (Catania 2005; Dickman 1988; Ward et al. 1997). Similar to rodents, hedgehogs possess a vomeronasal organ (VNO) or Jacobson's organ, which plays a chemosensory role in sexual and territorial behaviours through pheromone detection (Døving and Trotier 1998; Kondoh et al. 2021; Wöhrmann‐Repenning 1984). VNO dendrites project into the lumen thanks to their knobs, while the axons enter the vomeronasal soft tissue and constitute the nerve bundles that enter the olfactory bulb. The olfactory bulb is composed of the main olfactory bulb, which receives the olfactory nerve from the olfactory epithelium, and the dorsal accessory olfactory bulb, which receives the vomeronasal nerves (López‐Mascaraque et al. 1986). Most mammals have lateral nasal glands (LNG) or Steno's glands, which produce odorant binding proteins (Chamanza and Wright 2015; Nowack and Wöhrmann‐Repenning 2011). These proteins are excreted in the rostral nasal cavity via the LNG duct, where they capture chemical scent stimuli and present them to olfactory or VNO neurons (Pevsner and Snyder 1990). The olfactory lamina propria additionally contains scattered Bowman's glands, which secrete sulphomucins that form the extracellular component of the olfactory epithelium in rodents (Cuschieri and Bannister 1974). Apart from olfaction, the nose of hedgehogs also plays a tactile function via several whiskers and numerous microvibrissae arising from the integument of the ventrolateral glabrous rhinarium (Catania et al. 2000). Hedgehogs breathe predominantly through their nose (Johnson 2011), which directs, warms and moistens incoming air via turbinates and their associated vasculature. The nasal mucosa forms an important initial line of defence in preventing pathogens and harmful inhalants from reaching the deeper airways, predominantly via mucociliary clearance but also via secretion of antimicrobial and immunomodulating peptides such as defensins (Chamanza and Wright 2015; Lillard Jr et al. 1999; Zhang et al. 2016). Despite the crucial role of the olfactory system and upper respiratory tract for communication and survival, anatomical descriptions of the nasal cavity in this species are limited to several older documents, some of which are difficult to access (Fawcett 1918; Michelsson 1922; Wöhrmann‐Repenning 1975, 1984). The aim of this study is to describe the histological and anatomical features of the normal nasal cavity in the European hedgehog at fixed landmarks, which could act as a foundation for future studies investigating upper respiratory tract physiology or disease.

## 2 Materials and Methods

Five European hedgehogs (2 juveniles, 3 adults; 3 males, 2 females) admitted to wildlife rehabilitation centres in Cheshire (UK) and without a history of respiratory clinical signs were included in the study. Age was estimated based on weight combined with dentition for juveniles and by counting periosteal growth lines in the mandible for adults (Rasmussen et al. 2023), which determined hedgehogs to be between 4 months and 6 years of age. Euthanasia was a clinical decision based on veterinary discretion and no animals were euthanised for the purpose of this study. Ethical approval was granted by the University of Liverpool (UoL) number VREC1373.

**FIGURE 1:**
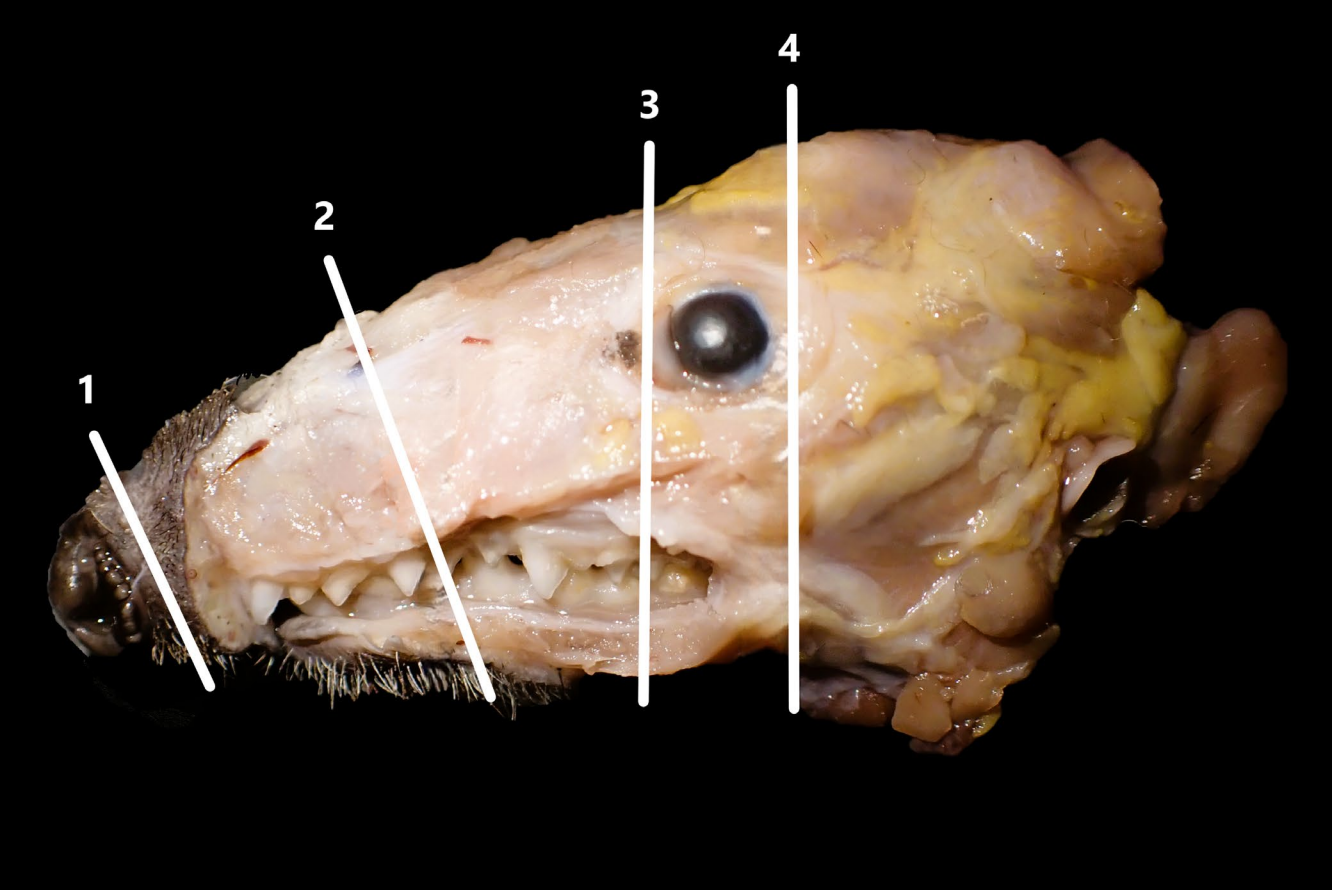
European hedgehog skull after formalin fixation and skin dissection. Four transverse sections of the nasal cavity (′Sections 1, 4′) were obtained at landmarks described in the text.

Four 5‐mm‐thick, transverse sections were obtained from the nasal cavity after ≥ 48 h of fixation in formalin (Figure 1). The first section (1) was obtained by cross‐sectioning the rhinarium, 5–10 mm from the tip of the snout. Additional sections were obtained with a diamond saw, at the first premolar (2), and at the rostral (3) and caudal aspect (4) of the eye. Tissue sections were placed in cassettes and underwent decalcification for 3–4 days at room temperature (formic acid, RDFTM, Cellpath) prior to embedding. Sections (4–5 μm) of paraffin‐embedded tissues were cut with a microtome, transferred onto glass slides and stained with haematoxylin and eosin (HE) for examination of histo‐anatomical features by light microscopy. Periodic acid‐Schiff (PAS) and Alcian blue stains were performed to better characterise intranasal glands and goblet cells. Glands were considered ‘serous' when PAS positive/Alcian blue negative, 'mucous' when Alcian blue positive/PAS negative and ‘mucoserous’ when positive for both (Cuschieri and Bannister 1974).

## 3 Results

**FIGURE 2:**
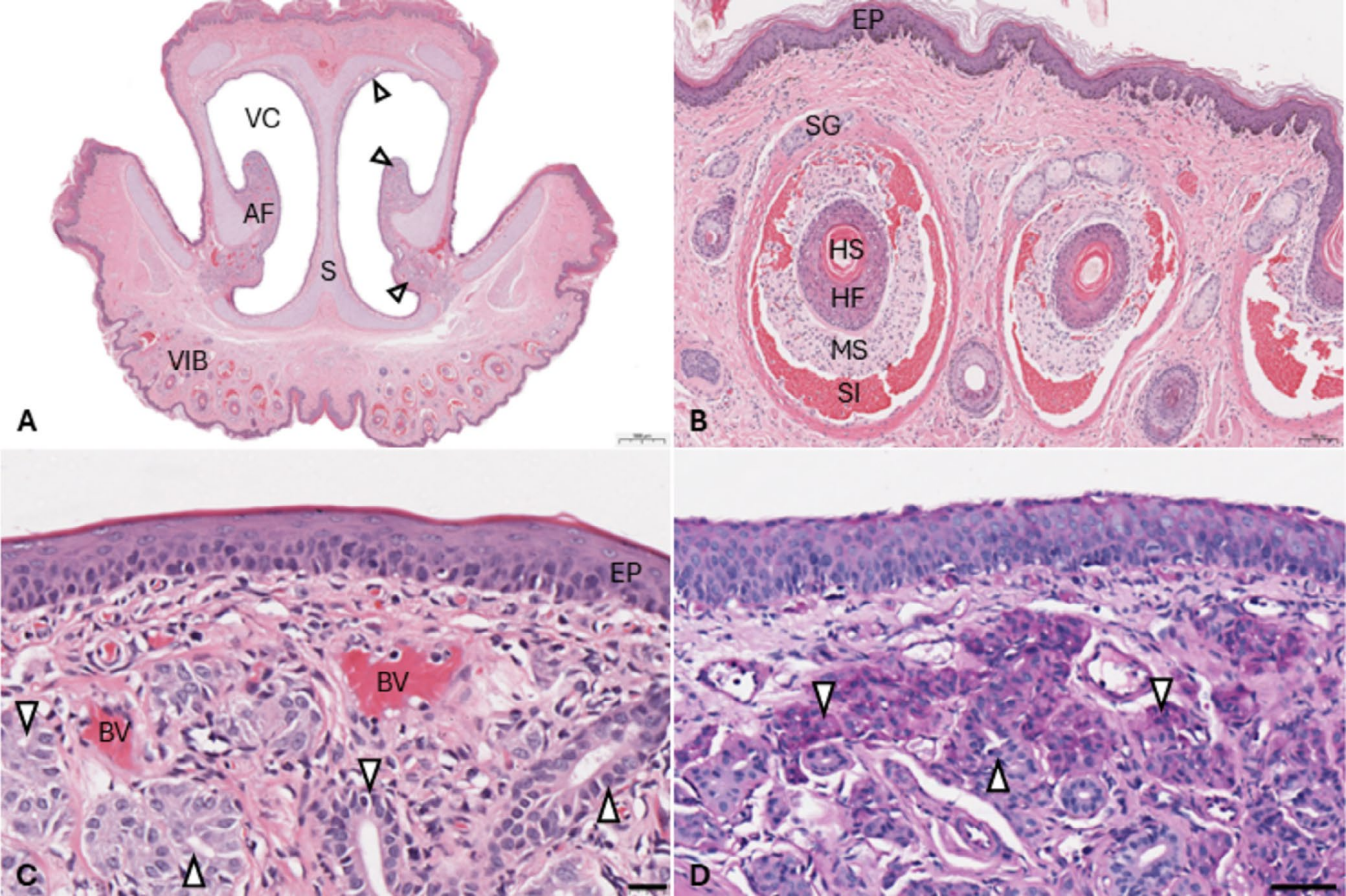
Histo‐anatomy of the vestibule in the European hedgehog. (A) Complete transverse section at ′Section 1′ (see Figure 1). AF, Alar folds; VC, vestibular cavity containing abundant serous glands (arrow heads). VIB, Vibrissae. Bar = 1 mm. H&E. (B) Vibrissae comprise a central hair shaft (HS) supported by the hair follicle (HF), mesenchymal sheath (MS) with nerve bundles and blood‐filled sinus (SI) surrounded by a capsule containing several apical sebaceous glands (SG). Bar = 100 μm. H&E. (C) Keratinised vestibular epithelium (EP) at the alar folds with subepithelial glands (arrow heads) and blood vessels (BV). Bar = 20 μm. H&E. (D) Serous glands (arrow heads) within the alar fold. Bar = 60 μm. PAS.

Apart from mild to moderate variation in height and width of nasal cavities (i.e., maxillary profile), anatomical features were largely shared between all five animals in the study and are therefore described together. From rostral to caudal, the nasal cavity of the hedgehog comprises the vestibule, the ostium, the nasal chamber proper and the nasopharynx. The vestibule is approximately 1 mm in width at its narrowest point and characterised by ventrolateral alar folds, which are supported by a relatively thick cartilaginous core (Figure 2A,B). The alar folds contain abundant mucosal serous glands and blood vessels, with fewer individual serous glands located along the septum, especially dorsally (Figure 2A,C,D). The vestibule is lined by keratinised squamous epithelium, 4–6 cell layers thick. Melanocytes are regularly observed near the basement membrane, but goblet cells are absent. The integument at the ventral aspect of the vestibule contains numerous microvibrissae (18–24 per section) (Figure 2A,B). The LNG ducts exit into the lateral aspect of the rostral nasal cavity between Sections 1, 2 (not visible on images). Here, the atrioturbinates can be observed caudal to the alar folds.

**FIGURE 3:**
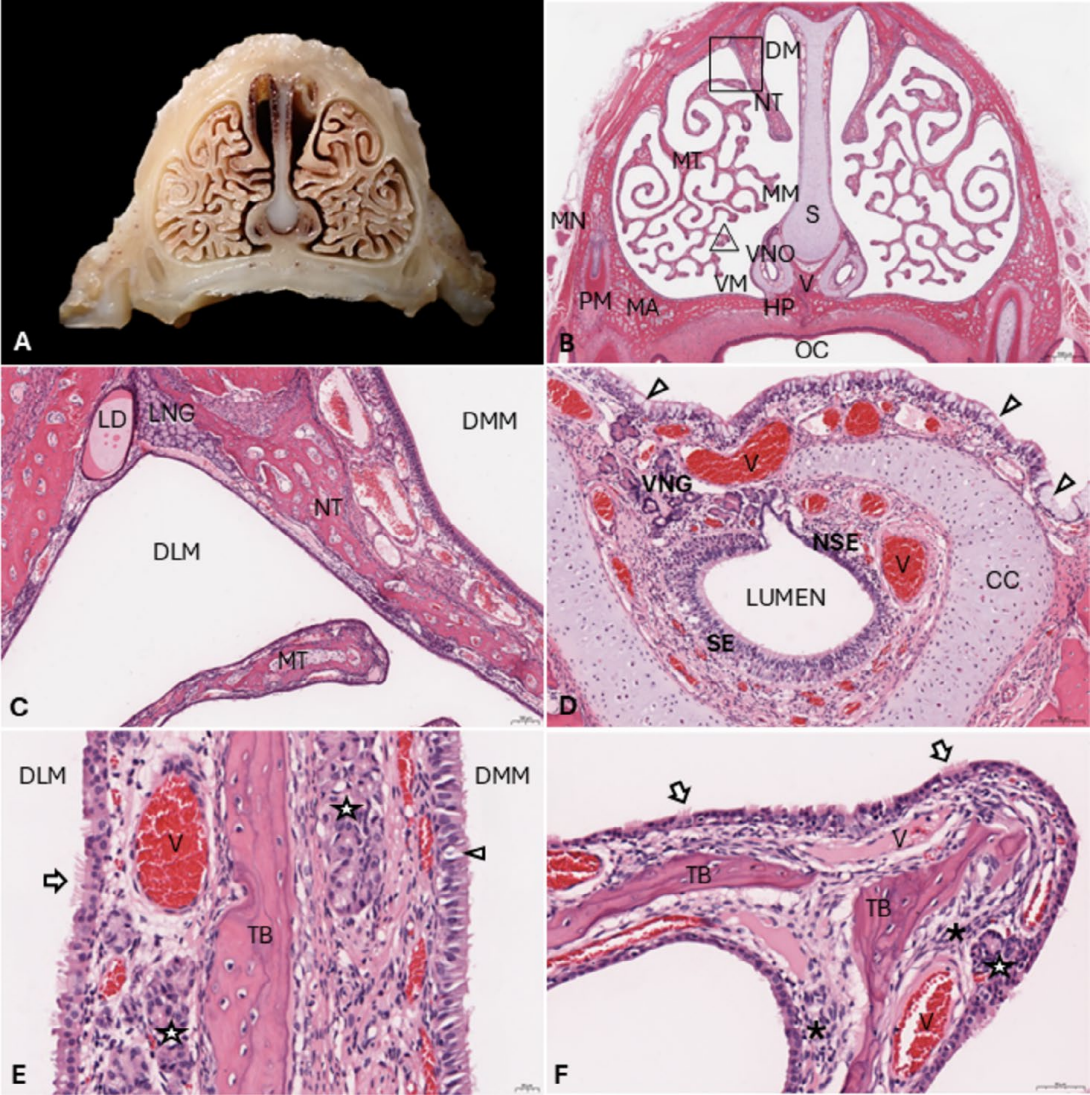
Nasal chamber proper in European hedgehogs (A) Gross morphology of ‘Section 2' (see Figure 1). (B) Corresponding histological section. DM, Dorsal meatus; HP, Hard palate; MA, Maxilla; MM, Medial meatus; MN, Maxillary (infraorbital) nerve; MT, Maxilloturbinates; NT, Nasoturbinates; OC, Oral cavity; PM, Premolar; S, Septum; V, Vomer; VM, Ventral meatus; VNO, Vomeronasal organ. Bar = 1 mm. H&E. (C) Close up of the dorsal meatus (square in Figure 3B). DLM, Dorsolateral meatus; DMM, Dorsomedial meatus; LD, Lateral nasal gland duct; LNG, Lateral nasal gland; rostral extension. Bar = 100 μm. H&E. (D) Vomeronasal organ with sensory epithelium (SE), nonsensory epithelium (NSE) and the VNO gland (VNG). CC, Cartilaginous capsule; V, Veins. Arrowheads point at goblet cells within the ventral nasal epithelium. Bar = 100 μm. H&E. (E) Nasoturbinate, higher magnification. Left, DLM; Right, DMM; Arrow, cilia; Arrowhead, goblet cell; stars, glands; TB, Turbinate bone lamella. Bar = 20 μm. H&E. (F) Maxilloturbinates (triangle in Figure 3B) lined by respiratory epithelium with veins (V), clusters of mesenchymal stromal cells (asterisks) and scattered glands (arrow). Bar = 50 μm. H&E.

The nasal chamber proper is divided into the left and right nasal cavities by a cartilaginous septum that widens towards the ventral aspect (Figure 3A,B). Hedgehogs have two types of turbinates in the nasal chamber proper: simple dorsal nasoturbinates (NT) and highly complex and branching maxilloturbinates (MT) that project from the lateral maxillae and extend throughout the nasal cavity (Figure 3A,B). The NT divides the dorsal meatus into the dorsomedial (DMM) and dorsolateral (DLM) meatus. The LNG duct (Figure 3C) is located within the mucosal lamina propria, lining the junction between the maxilla and NT, at the dorsolateral meatus and it is surrounded by numerous mucoserous glands, presumably rostral extensions from the LNG. NT (Figure 3E) are supported by 10‐ to 100‐μm‐thick lamellar bone and lined by columnar, frequently ciliated respiratory epithelium (RE) with 1–2 cell layers and between 20 (DLM) and 50 (DMM) μm thick. Goblet cells are rare in the DLM and sparsely present (1 per 15–30 epithelial cells) in the DMM for most animals, except for one adult, which had abundant (1 per 2–5 epithelial cells) DMM goblet cells. The DMM lamina propria contains relatively large blood vessels and several scattered mucous glands. MT (Figure 3F) are supported by 10‐ to 100‐μm thick, occasionally discontinuous, branching bone lamellae surrounded by a 40‐ to 60‐μm‐thick lamina propria containing numerous small‐ to medium‐sized blood vessels, rare serous glands, small numbers of resident lamina propria lymphocytes, occasional individual lamina propria, intraepithelial or intraluminal neutrophils (only for some animals and in < 20% of surface area), and prominent mesenchymal stromal cell clusters at the junction with the turbinate bone. Osteoclasts were occasionally observed in small numbers (0–2 per 10 high‐power fields; 1.52 mm^2^). Turbinate lamellae appeared thicker, especially distally, for adults compared to juveniles. The MT (Figure 3F) were predominantly lined by a single, or occasionally pseudostratified, partially ciliated cuboidal to columnar RE with regular basal nuclei. Goblet cells were rare within the MT epithelium, but they were present in substantially higher densities (1 per 1–5 epithelial cells) within the RE lining the ventrolateral maxilla and the ventral septum.

The hedgehog nasal chamber proper also contains the VNO (Figure 3B), a bilateral tubular structure located over the palate on the two sides of the nasal septum that is surrounded by a cartilaginous capsule. Rostrally, the VNO lies directly on top of the hard palate, whereas both structures are separated by the ventral meatus towards caudal. The vomeronasal lumen is lined by a sensory epithelium (SE) in the medial wall and by a nonsensory epithelium (NSE) in the lateral wall (Figure 3D). The VNO SE is a 60‐ to 90‐μm‐thick pseudostratified epithelium mainly composed of 2–3 layers of bipolar neurons. The VNO SE also includes the epithelial supporting cells in the superficial part, and basal cells deep to the neuronal layer. The NSE is a 15‐ to 40‐μm‐thick pseudostratified epithelium with nonciliated and ciliated cells. Small numbers of leukocytes, especially neutrophils, can be found within and directly below the NSE. In the vomeronasal soft tissue, beyond the nerve bundles, blood vessels and serous glands are present.

**FIGURE 4:**
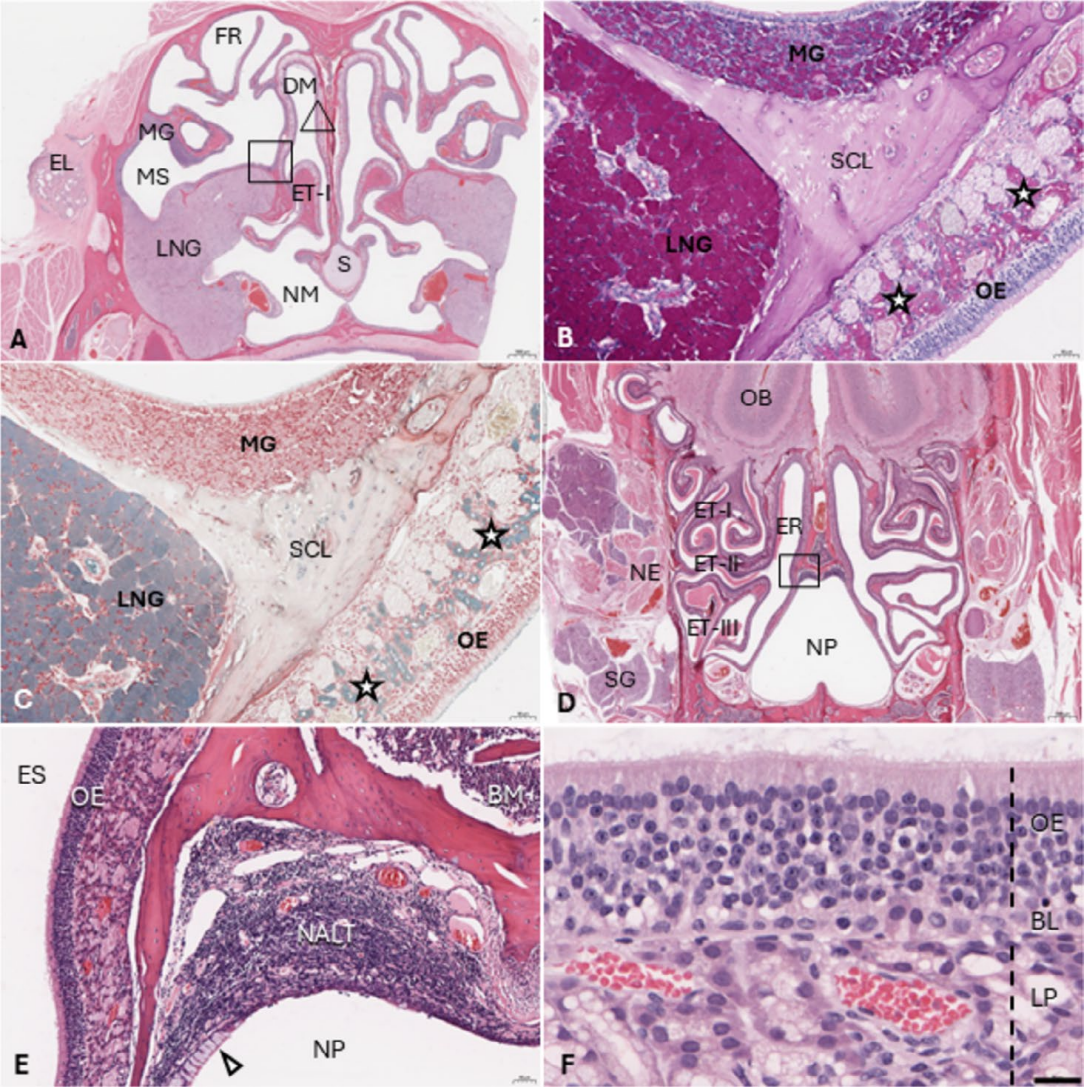
The deep nasal cavity (A) Histology of ′Section 3′ (see Figure 1). DM, Dorsal meatus of the nasal fossa; EL, Eyelid and tear duct; ET‐I, Ethmoturbinate I; FR, Frontal recess; LNG, Lateral nasal gland; MG, Maxillary gland; MS, Maxillary sinus; NM, Nasopharyngeal meatus; S, Septum. Bar = 1 mm. H&E. (B) PAS stain highlighting the LNG, MG and Bowman's glands (stars) within the olfactory mucosa of the ethmoid sinus (ES). Corresponds to the square in Figure 4A. Bar = 50 μm. SCL, Semicircular lamina of the caudal nasoturbinate. (C) Alcian blue stain highlighting the LNG and Bowman's glands (stars) throughout the olfactory lamina propria. Bar = 50 μm. (D) Histology of the nasopharynx (NF) and olfactory bulb (OB) (Figure 1, ′Section 4 ′). ET‐I‐III, ethmoid turbinates; ER, ethmoid recess; NE, nerves; SG, salivary gland. Bar = 1 mm. H&E. (E) Dorsal NP (rectangle Figure 4D) with nasopharynx‐associated lymphoid tissue (NALT) and goblet cells (arrow heads) in the adjacent epithelium. BM, Bone marrow. Bar = 50 μm. H&E. (F) Olfactory epithelium (triangle Figure 4A) lining most of the dorsocaudal nasal cavity and sinuses. BL, Basal layer; LP, Lamina propria. Bar = 20 μm. H&E.

The left and right nasal cavities merge rostral to the nasopharynx, as the septum becomes discontinuous at the ventral aspect (Figure 1 ‘Section 3', Figure 4A). The LNG is characterised by numerous mucoserous acini and several striated ducts with a single layer of columnar epithelium, and occupies approximately one third of the section at this location. The more caudal serous maxillary gland is much smaller, containing few inconspicuous nonstriated ducts and being mostly confined to the lateral maxillary sinus but extending medially via the semicircular lamina of the caudal nasoturbinate (Figure 4A–C). The maxillary sinus can be appreciated better just caudal to Section 3 (Supplement 1). The frontal recess is separated dorsoventrally into two cavities by the frontal lamina, and the dorsal part is lined by the caudal aspect of the maxilla, right before the latter transitions into the frontal bone. Section 3 also contains the rostral aspect of ethmoturbinate I, which extends into the ethmoturbinate recess and forms the ethmoid labyrinth caudally, situated between both eyes. Several scattered mucoserous acinous Bowman's glands are present within the lamina propria of the ethmoid sinuses and the dorsal half of the septum, the secretions of which support the olfactory epithelium. The epithelium lining the caudoventral nasal cavity and the ventral aspect of the septum is characterised by a single layer (30–50 μm) of ciliated columnar cells containing numerous goblet cells and a single continuous layer of basal cells. The entire dorsal caudal nasal cavity, as well as the middle and dorsal aspects of the septum, are lined by olfactory epithelium, 6–9 cell layers (60–80 μm) thick with granular cytoplasm and cilia, and basal nuclei with fine to finely stippled chromatin and up to two densely basophilic nucleoli. Goblet cells are absent throughout the olfactory epithelium (Figure 4F).

The most caudal nasal cavity section (Figure 1, ‘Section 4') comprises the olfactory bulbs, the ethmoturbinates, ethmoid recess and an inverted heart‐shaped nasopharynx with dorsal nasopharyngeal‐associated lymphoid tissue (NALT) (Figure 4D,E). Three ethmoturbinates project from the ethmoid recess on this section, referred to as turbinates I–III, ordered from dorsal to ventral and all lined entirely by olfactory epithelium (50–100 μm). The nasopharynx, with the exception of the NALT, is lined almost entirely by respiratory epithelium (30–50 μm) with abundant goblet cells. Epithelium overlying the NALT is of lesser thickness (5–20 μm) with occasional cilia (Figure 4E).

## 4 Discussion

Similar to rodents and dogs, hedgehogs have extensive olfactory epithelium, complex maxilloturbinates, accessory olfactory organs such as the VNO, relatively large LNG and olfactory bulbs, suggesting that olfaction is an important function of the nasal cavity (Catania et al. 2000; Chamanza and Wright 2015; Kondoh et al. 2021; López‐Mascaraque et al. 1986; Wöhrmann‐Repenning 1984). With the exception of *Tupaia belangeri* , the complexity of hedgehog MT is contrary to the simpler MT seen in moles and other shrew species, despite phylogenetic relatedness (Feng et al. 2022; Ito et al. 2022; Wöhrmann‐Repenning 1975). The 1st premolar section ('Section 2') provided a good overview of the MT. Therefore, this section could be a suitable reference point to assess turbinate atrophy in hedgehogs, just like in pigs (Martineau‐Doizé et al. 1991). Section 3 appears useful to assess the LNG, maxillary sinus and frontal recess, whereas Section 4 could be used to assess NALT and the ethmoturbinates.

The anatomy of the VNO confirms what was previously described in *E. europaeus* (Wöhrmann‐Repenning 1984) and other hedgehog species (Kondoh et al. 2021), supporting its role in the intra‐ and interspecific chemical communication of the European hedgehog. Semiochemical detection occurs in the vomeronasal SE, in which bipolar neurons detect molecules in the VNO lumen and project signals to the AOB via the vomeronasal nerves (Salazar and Sánchez Quinteiro 2009). In rats and shrews, the LNG excretory duct is located ventral to the nasoturbinates and exits in the vestibulum (Moe and Bojsen‐Moller 1971; Nowack and Wöhrmann‐Repenning 2011), similar to our observation in *E. europaeus* . In other mammals, the LNG is usually described predominantly as a serous gland that produces a watery film (Chamanza and Wright 2015; Nowack and Wöhrmann‐Repenning 2011). In contrast, the hedgehog LNG also has a moderately intense Alcian blue positive staining (Figure 4C), suggesting a mucoid component. The two distinctly different staining properties (light vs. densely eosinophilic) observed within the LNG duct (Figure 3C) on H&E stain may further support a dual composition of hedgehog LNG secretions. The mucous layer that covers the apical side of the nasal epithelium is predominantly produced by goblet cells and lamina propria glands (Zhang et al. 2016). In European hedgehogs, goblet cells were abundant at the maxillary, septal and nasopharyngeal respiratory epithelium. Bowman's glands likely play an important role in mucous production for the dorsocaudal nasal cavity and ethmoid sinuses.

There were slight variations in morphology between sections from different animals, especially the height‐to‐width ratio of the maxilla. This could be due to individual variables (sex, age), variation in section locations or genetic and geographical variations in skull morphology. Additionally, mild to moderate differences in goblet cell density were noted, mostly in the DMM. Based on the increased thickness of the epithelium in the DMM compared to the DLM, ‘Section 2’ may correspond to an area of natural transition from respiratory to olfactory epithelium, in which case a few millimetres of difference in depth during sectioning or trimming may result in variable histological features. Epithelial metaplasia and goblet cell hyperplasia are also physiological adaptations to damage (Chamanza and Wright 2015). The rodent DMM seems predisposed to indirect damage from several secondary toxic compounds because of a combination of metabolising enzymes and accumulation of relatively high concentrations of inhaled substances (Robinson et al. 2003). It is therefore possible that the hedgehogs with increased goblet cells were previously exposed to irritants. A limitation of this study is the lack of longitudinal or dorsoventral sections, which would have enabled additional descriptions regarding transition zones of different epithelial and glandular types (Chamanza and Wright 2015). Supportive imaging techniques, such as a CT scan, could have been helpful to corroborate histo‐anatomical findings, but these are described in other studies (Ruszkowski et al. 2024). Classifying glands according to PAS and Alcian blue staining properties provides only a superficial level of assessment of secretory components, which may vary between locations despite staining similarities, as has been demonstrated in other species (Cuschieri and Bannister 1974; Musa et al. 2012). Additional histochemistry and immunohistochemistry are therefore warranted.

Finally, there is some discrepancy between studies regarding the terminology for the sinuses in *E. europaeus* . According to Wöhrmann‐Repenning (1975), the only pneumatic cavity in insectivores is the maxillary sinus. However, the frontal recess is also well described in the previous study, and Ruszkowski et al. (2024) briefly mention a frontal sinus in the species. Studies in shrews describe a dorsal frontal recess that is divided into different compartments by frontal turbinates, and a lateral maxillary sinus that is partially embedded by the maxilla and drains rostrally into the nasal cavity, but there is no mention of a frontal sinus (Feng et al. 2022; Ito et al. 2022; Nowack and Wöhrmann‐Repenning 2011). Our findings are comparable with those previously described in *E. europaeus* (Wöhrmann‐Repenning 1975), with several similarities in shrews and moles; therefore, consistency with this terminology is favoured (Figure 4A).

This study described the histoanatomical features of the nasal cavity and associated sensory structures in European hedgehogs based on four standardised sections. The nasal cavity is a functionally important organ in the species. Although clinical respiratory disease is common in hedgehogs, this is often attributed to lung conditions such as verminous pneumonia (Bexton 2016; Van de Weyer et al. 2023). However, diagnostic investigations of the nasal cavity rarely take place because of various limitations, including a lack of histoanatomical references. This descriptive work may guide diagnostic investigations and future research exploring upper respiratory tract function and disease in European hedgehogs.

## Conflicts of Interest Statement

The authors declare no conflicting interests. All authors agreed to publish this work.

## Supporting information


**Appendix S1:** Supporting Information.

**Appendix S2:** Supporting Information.

## Data Availability

The data that support the findings of this study are available from the corresponding author upon reasonable request.
