# Supplementary material for: Descriptive Histology and Anatomy of the Nasal Cavity and Its Associated Sensory Organs in the European Hedgehog ( Erinaceus europaeus ) Based on Four Standardised Transverse Sections
Source: Anat Histol Embryol. 2025 Oct 19;54(6):e70067. doi: 10.1111/ahe.70067 (PMC12535753; doi:10.1111/ahe.70067)
Supplement: Supplementary file 1 — Appendix S1: Supporting Information. [file AHE-54-e70067-s002.docx]

Supplement 1: Additional sections of the caudal nasal cavity from rostral to caudal. A) Caudal nasal chamber proper, obtained rostral to ‘section 3’. B) Section 3. The left and right ventral nasal cavities merged into the nasopharynx. C) Section containing the maxillary sinus (arrow) and the caudal frontal recess. D) Section rostral to ‘section 4’, containing the ethmoid labyrinth (arrowhead), ethmoid recess and nasopharynx (star).
