# Supplementary figures and images for: Descriptive Histology and Anatomy of the Nasal Cavity and Its Associated Sensory Organs in the European Hedgehog ( Erinaceus europaeus ) Based on Four Standardised Transverse Sections
Source: Anat Histol Embryol. 2025 Oct 19;54(6):e70067. doi: 10.1111/ahe.70067 (PMC12535753; doi:10.1111/ahe.70067)

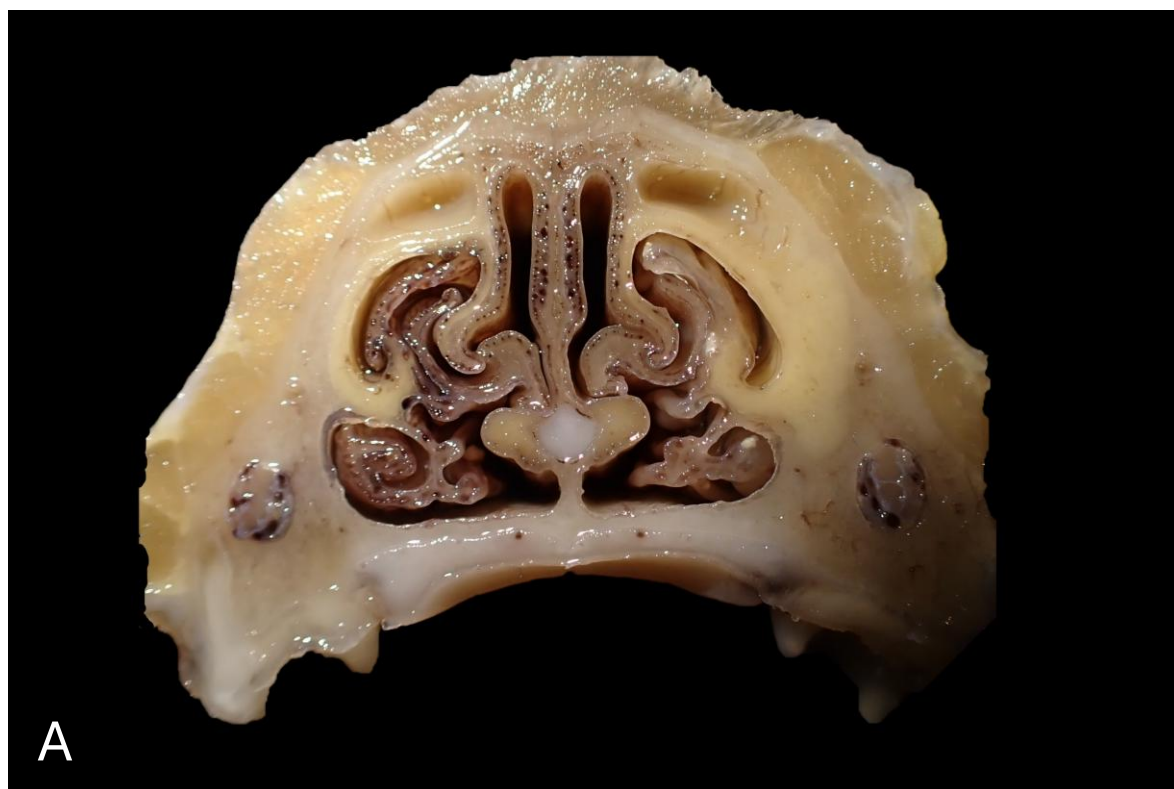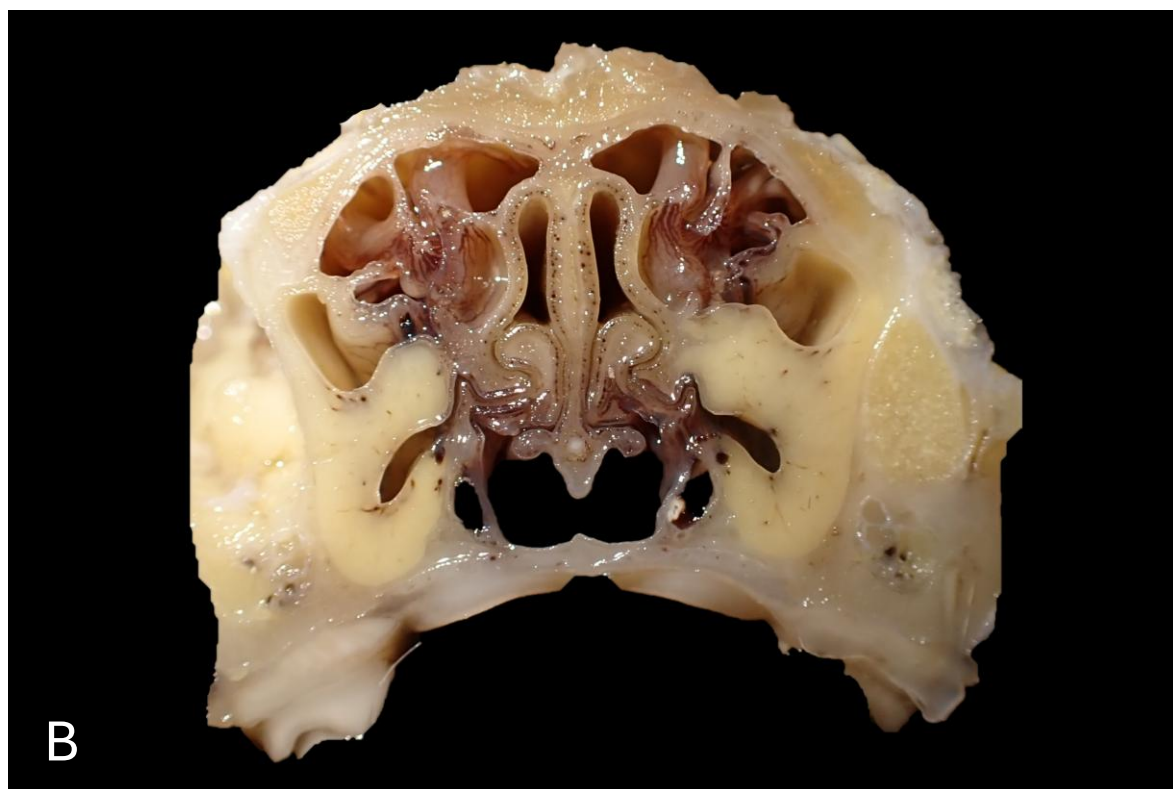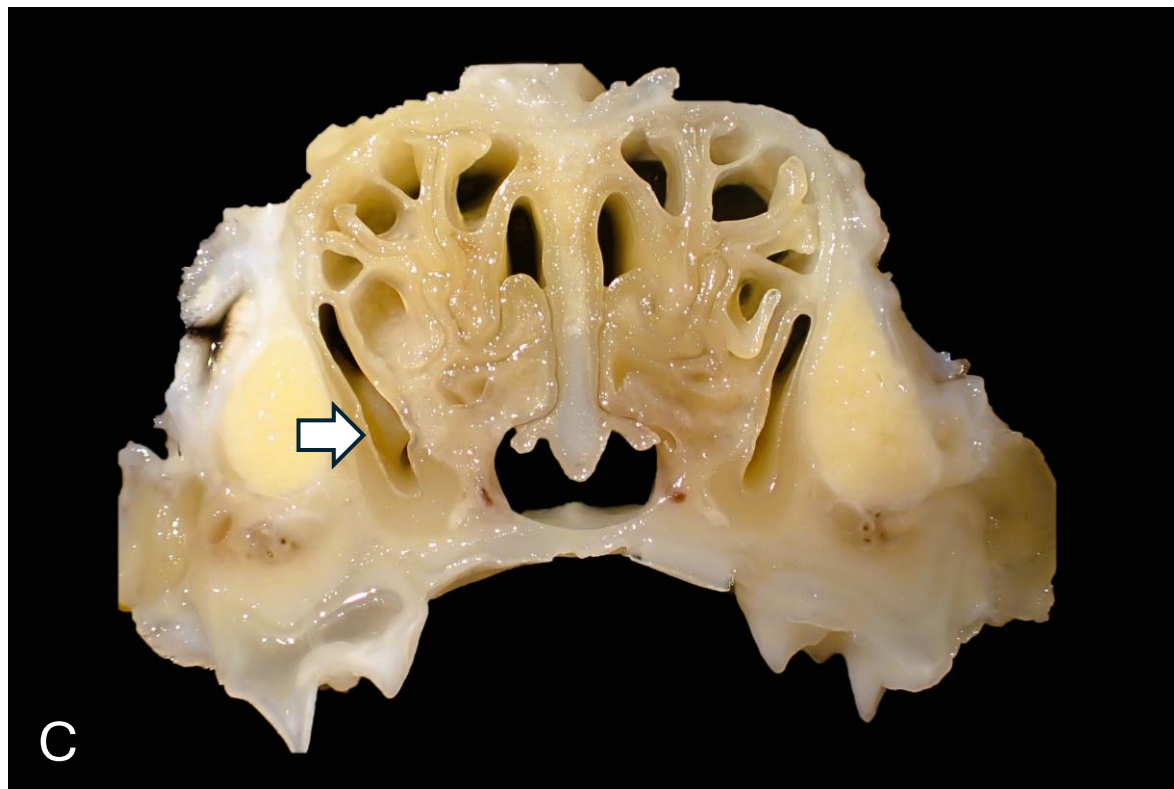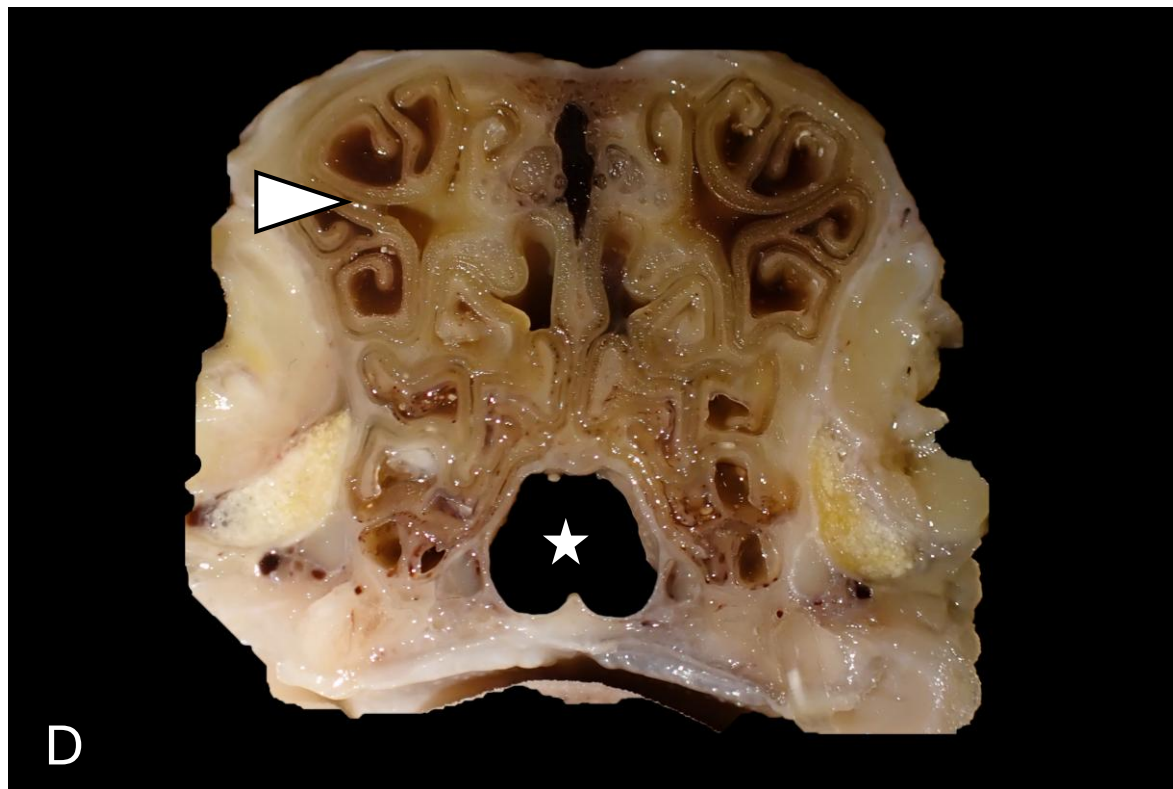

Supplement: Supplementary file 2 — Appendix S2: Supporting Information. [file AHE-54-e70067-s001.pdf]
